# Supplementary material for: Microtubules in the axon are GDP bound but adopt a stable GTP-like expanded state
Source: Nat Struct Mol Biol. 2026 Apr 8;33(4):631–40. doi: 10.1038/s41594-026-01787-7 (PMC13095656; doi:10.1038/s41594-026-01787-7)
Supplement: Supplementary file 1 — Supplementary Fig. 1 and Table 1. [file 41594_2026_1787_MOESM1_ESM.pdf]

nature structural & molecular biology

Supplementary information https://doi.org/10.1038/s41594-026-01787-7

Microtubules in the axon are GDP bound but adopt a stable GTP-like expanded state

In the format provided by the authors and unedited

Supplementary Table 1. Dimer twist of microtubules in different states

MT states	Dimer Twist (degrees)	Reference
Axonal 13 PF	0.09	EMD-70956
Brain-GMPCPP 13 PF	0.23	[10]
Brain-GDP 13 PF	0.08	[10]
E254A-GTP 13PF	-0.22	EMD-25158[6]

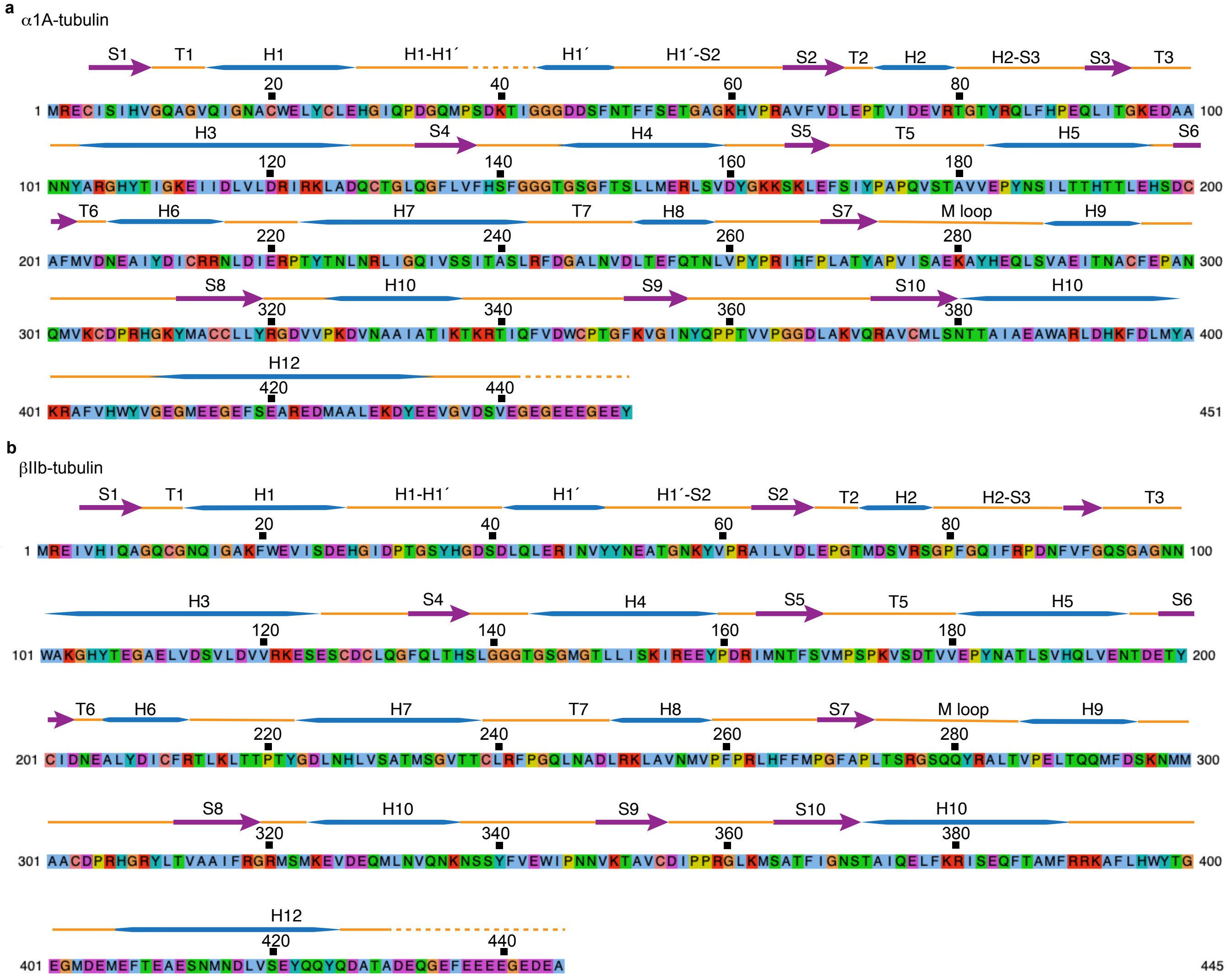

Supplementary Figure 1. Sequences of human α1A- and βIIb-tubulin isoforms. β-strands, purple arrows; helices, blue lines; loops orange lines.
