# Supplementary material for: Microtubules in the axon are GDP bound but adopt a stable GTP-like expanded state
Source: Nat Struct Mol Biol. 2026 Apr 8;33(4):631–40. doi: 10.1038/s41594-026-01787-7 (PMC13095656; doi:10.1038/s41594-026-01787-7)
Supplement: Supplementary file 2 — Reporting Summary [file 41594_2026_1787_MOESM2_ESM.pdf]

nature portfolio

Corresponding author(s): Dr. Antonina Roll-Mecak

Last updated by author(s): Jul 7, 2025

Reporting Summary

Nature Portfolio wishes to improve the reproducibility of the work that we publish. This form provides structure for consistency and transparency in reporting. For further information on Nature Portfolio policies, see our Editorial Policies and the Editorial Policy Checklist.

Statistics

For all statistical analyses, confirm that the following items are present in the figure legend, table legend, main text, or Methods section.

n/a	Confirmed	
☐	☒	The exact sample size (*n*) for each experimental group/condition, given as a discrete number and unit of measurement
☐	☒	A statement on whether measurements were taken from distinct samples or whether the same sample was measured repeatedly
☐	☒	The statistical test(s) used AND whether they are one- or two-sided<br>*Only common tests should be described solely by name; describe more complex techniques in the Methods section.*
☒	☐	A description of all covariates tested
☒	☐	A description of any assumptions or corrections, such as tests of normality and adjustment for multiple comparisons
☐	☒	A full description of the statistical parameters including central tendency (e.g. means) or other basic estimates (e.g. regression coefficient) AND variation (e.g. standard deviation) or associated estimates of uncertainty (e.g. confidence intervals)
☒	☐	For null hypothesis testing, the test statistic (e.g. *F*, *t*, *r*) with confidence intervals, effect sizes, degrees of freedom and *P* value noted<br>*Give P values as exact values whenever suitable.*
☒	☐	For Bayesian analysis, information on the choice of priors and Markov chain Monte Carlo settings
☒	☐	For hierarchical and complex designs, identification of the appropriate level for tests and full reporting of outcomes
☒	☐	Estimates of effect sizes (e.g. Cohen's *d*, Pearson's *r*), indicating how they were calculated

Our web collection on statistics for biologists contains articles on many of the points above.

Software and code

Policy information about availability of computer code

Data collection	Serial EM v4.1
Data analysis	Cryosparc v4, FREALIGN v9.11, ISOLDE v1.6, Coot v0.9.8.91, PHENIX v1.20.1_4487, ChimeraX-1.6.1, Dynamo v1.1.546, IMOD v5.1.0, EMAN2 v2.99, DeepEMhancer v0.15, Subtom v1.16-32f731b

For manuscripts utilizing custom algorithms or software that are central to the research but not yet described in published literature, software must be made available to editors and reviewers. We strongly encourage code deposition in a community repository (e.g. GitHub). See the Nature Portfolio guidelines for submitting code & software for further information.

Data

Policy information about availability of data

All manuscripts must include a data availability statement. This statement should provide the following information, where applicable:

- Accession codes, unique identifiers, or web links for publicly available datasets
- A description of any restrictions on data availability
- For clinical datasets or third party data, please ensure that the statement adheres to our policy

Cryo-EM maps and atomic model are deposited at the EMDB and PDB with accession numbers: EMD-70956 an 9OX7, respectively, with accompanying raw half maps. Scripts used for power spectrum analysis were deposited on Github (https://github.com/RollmecakLab/Layer-Line-Analysis)

Research involving human participants, their data, or biological material

Policy information about studies with human participants or human data. See also policy information about sex, gender (identity/presentation), and sexual orientation and race, ethnicity and racism.

Reporting on sex and gender	n/a
Reporting on race, ethnicity, or other socially relevant groupings	n/a
Population characteristics	n/a
Recruitment	n/a
Ethics oversight	n/a

Note that full information on the approval of the study protocol must also be provided in the manuscript.

Field-specific reporting

Please select the one below that is the best fit for your research. If you are not sure, read the appropriate sections before making your selection.

☒ Life sciences ☐ Behavioural & social sciences ☐ Ecological, evolutionary & environmental sciences

For a reference copy of the document with all sections, see nature.com/documents/nr-reporting-summary-flat.pdf

Life sciences study design

All studies must disclose on these points even when the disclosure is negative.

Sample size	No sample size calculation was performed. For the layer line analyses conducted in both in vitro and in situ conditions, tens of tomograms were collected, encompassing a broad sampling of microtubules across multiple datasets. The majority of microtubules present within these tomograms were analyzed, providing a sufficiently large and representative dataset to support robust conclusions.
Data exclusions	Single-particle cryo-EM images with a resolution less than than 7 Å were excluded. Tomograms with low signal to noise ratio or with misaligned frames were excluded.
Replication	n/a
Randomization	n/a
Blinding	n/a

Reporting for specific materials, systems and methods

We require information from authors about some types of materials, experimental systems and methods used in many studies. Here, indicate whether each material, system or method listed is relevant to your study. If you are not sure if a list item applies to your research, read the appropriate section before selecting a response.

Materials & experimental systems

n/a	Involved in the study	
☒	☐	Antibodies
☐	☒	Eukaryotic cell lines
☒	☐	Palaeontology and archaeology
☒	☐	Animals and other organisms
☒	☐	Clinical data
☒	☐	Dual use research of concern
☒	☐	Plants

Methods

n/a	Involved in the study	
☒	☐	ChIP-seq
☒	☐	Flow cytometry
☒	☐	MRI-based neuroimaging

Eukaryotic cell lines

Policy information about cell lines and Sex and Gender in Research

Cell line source(s)	Induced pluripotent stem cells (iPSCs) expressing the neuronal transcriptional activator neurogenin 2 (NGN2) under the control of a doxycycline-inducible promoter were obtained from Michael E. Ward lab, and generated as previously described (Wang C et al. Stem Cell Reports. 2017; 9: 1221-1233; Fernandopulle MS et al. Curr Protoc Cell Biol. 2018; 79: e51). Sex-male.
Authentication	Cell were authenticated by PCR
Mycoplasma contamination	The cell lines tested negative for Mycoplasma. Mycoplasma free cell culture was assessed by the absence of DAPI staining in the cytoplasm of cells.
Commonly misidentified lines (See ICLAC register)	These cell lines are not in the database.

Plants

Seed stocks	n/a
Novel plant genotypes	n/a
Authentication	n/a
